# Supplementary material for: Exploring variations in the implementation of a health system level policy intervention to improve maternal and child health outcomes in resource limited settings: A qualitative multiple case study from Uganda
Source: PLOS Glob Public Health. 2024 Nov 26;4(11):e0003290. doi: 10.1371/journal.pgph.0003290 (PMC11594426; doi:10.1371/journal.pgph.0003290)
Supplement: Attachment — Submitted filename: Response to Reviewers.docx [file pgph.0003290.s002.docx]

**DAVID ROGER WALUGEMBE, (PhD)**

**1223 Park Drive, Vancouver; Tel. 519 317-8481; +256 782 243477**

**E-mail :** [**dwalugembe@gmail.com/**](mailto:dwalugembe@gmail.com/) [**david.walugembe@ubc.ca**](mailto:david.walugembe@ubc.ca)

September 4, 2024

Dear Dr. Hoemeke,

**Re: Response to Reviewer’s feedback on our manuscript GPH-D-24-01046 titled**

**Exploring variations in the implementation of a health system level policy intervention to improve maternal and child health outcomes in resource limited settings: A qualitative multiple case study from Uganda”**

We acknowledge receipt of and thank you for the valuable feedback, and the points raised by the editor and reviewers. As required, please find below our responses to each of these points. A marked-up copy of our manuscript that highlights changes made to the original version has been uploaded as a separate file labeled ‘Revised Manuscript with track changes’. Additionally, an unmarked version of our revised paper without tracked changes has also been uploaded as a separate file labeled ‘Manuscript’. We have also uploaded the completed version of PLOS’ questionnaire on inclusivity in global health research.

First, we appreciate the editor and both reviewers for their feedback regarding the timely nature of our manuscript and our unique approach to policy analysis.

Secondly, we acknowledge the observation by reviewer 2 and the editor regarding the use of different terms i.e. health facilities, facilities, cases which could indeed be confusing and acknowledge the recommendation to use on term- “health facilities”. We reviewed the manuscript and found that we used the term facilities 31 times throughout the manuscript. For nine (9) out of the 31 times, we did not qualify the usage of the term. Subsequently, we have qualified eight (08) of these by adding ‘health’ before facilities as indicated in the version with track changes. In one instance, (page 23, line 753), we have changed the use of the term ‘facilities’ to cases. For additional contextual clarity, three of the nine times that the term ‘facilities’ was used without qualifying it were in the verbatim quotes which probably depicts its generally accepted usage within the study context to collectively refer/imply health facilities that are representative of the various levels of care as articulated in detail under the materials and methods section specifically on pages 7-8, lines 243-249.

We also observe that the use of the term ‘cases’ is congruent with the study design that we adopted for our study- multiple case study design. As explained under the materials and methods section on page 7, lines 242- 249, the unit of analysis (case) in this study was a health facility. However, this study involved various types and levels of health facilities including health center III, IVs, general and regional referral hospitals. Given the variations in the types of health facilities and levels of care included in our study, we prefer to maintain the usage of both terms – cases and health facilities because the context and usage varies and is congruent with the adopted study design. Altering incidences where we have used the term cases to health facilities may obscure comprehension and appreciation of the variations in the types of health facilities and levels of care under study. However, as recommended, to enhance clarity, we have eliminated the usage of the term ‘site/sites’ that appeared seven times across the manuscript and replaced it with ‘cases’ as indicated in version with track changes.

We acknowledge the suggestion to use key informants instead of study participants, however, given that our purposive sampling approach enabled us to recruit participants with expert knowledge and those that had varying lived experiences around its implementation, we prefer to collectively refer to all of them as study participants and not key informants. Our decision is partially informed by insights gleaned from Pahwa et al (2023) regarding usage of the term key informants in applied qualitative research accessible here <https://www.ncbi.nlm.nih.gov/pmc/articles/PMC10666509/>

Our third response is in relation to the observed need for “a description of the MPDSR policy”. We acknowledge this feedback and also note that our approach to describing the policy takes into consideration the need to inform the readers about its constituent parts including maternal and perinatal death audit/reviews (lines 185-188), its origin, evolution and purpose (lines 188-199) as well as benefits (lines 201-202). This approach resonates with the WHO description of the policy that highlights what the policy seeks to accomplish – <https://www.who.int/teams/maternal-newborn-child-adolescent-health-and-ageing/maternal-health/maternal-and-perinatal-death-surveillance-and-response>

We also agree with the observation that we do not describe the adoption of the policy in Uganda. This is true and it is beyond the scope of our manuscript which focused on reporting key findings regarding the observed variations in the implementation of the policy across selected health facilities and not the entire country. Our study was limited to selected health facilities and may not offer a true representation of the variations across the entire country. It is for this reason that upon acknowledging that Uganda’s gains in reducing maternal and neonatal morality have reportedly been partially attributed to the concerted efforts invested in the implementation of the MPDSR policy (page 6-7- lines 210-220), we dived in and focused on the aim of our study. Furthermore, in the detailed larger study (reference 50) accessible via this link <https://ir.lib.uwo.ca/etd/8204>, we describe how the differences in the sense making, relational, operational and appraisal efforts across the various cases (health facilities) may account for the observed variations in the implementation of the MPDSR policy across the selected cases/health facilities.

Thank you to reviewer 1 for the spot on feedback regarding the intents of our manuscript, the acknowledged study limitations and importance of the themes that we presented in our manuscript. We also acknowledge the encouraging feedback from reviewer 2 and their acknowledgment of the difficulties involved in separating the description of the process of implementing the policy from describing the implementation of the recommendations of the policy. This complexity arises in part from the fact that MPDSR seeks to use lessons learned to avoid the deaths of mothers and babies and thus separating the description of implementation process from the implementation of the recommendations is complex. Complex. However, guided by NPT, our approach explored the sense making, relational, operational and appraisal efforts of the people involved. Under each of these constructs, and their respective subconstructs, we explored what they do to implement the various components of the MPDSR including response. We also explored the barriers encountered and described these in detail under reference 50.

Journal requirements

As recommended, we reviewed our reference list and ensured that it is complete and correct. We do not have any studies/papers that have been retracted. No changes have been made to our reference list.

Sincerely,

David Roger Walugembe (Ph.D.)

Corresponding Author

Faculty of Medicine

Department of Anesthesiology, Pharmacology & Therapeutics

The University of British Columbia,
Vancouver, BC Canada V5Z 1M9
[david.walugembe@ubc.ca/dwalugembe@gmail.com](mailto:david.walugembe@ubc.ca/dwalugembe@gmail.com)

Tel: 519 317-8481

ORCID iD: (<https://orcid.org/0000-0001-5752-8656>)
